# A Televised, Web-Based Randomised Trial of an Herbal Remedy (Valerian) for Insomnia

**DOI:** 10.1371/journal.pone.0001040

**Published:** 2007-10-17

**Authors:** Andrew D. Oxman, Signe Flottorp, Kari Håvelsrud, Atle Fretheim, Jan Odgaard-Jensen, Astrid Austvoll-Dahlgren, Cheryl Carling, Ståle Pallesen, Bjørn Bjorvatn

**Affiliations:** 1 Norwegian Knowledge Centre for the Health Services, Oslo, Norway; 2 Norwegian Competence Center for Sleep Disorders, Haukeland University Hospital, Bergen, Norway; 3 Department of Psychosocial Science, University of Bergen, Bergen, Norway; 4 Section for General Practice, University of Bergen, Bergen, Norway; University of Toronto, Canada

## Abstract

**Background:**

This trial was conducted as part of a project that aims to enhance public understanding and use of research in decisions about healthcare by enabling viewers to participate in research and to follow the process, through television reports and on the web. Valerian is an herbal over-the-counter drug that is widely used for insomnia. Systematic reviews have found inconsistent and inconclusive results about its effects.

**Methods:**

Participants were recruited through a weekly nationally televised health program in Norway. Enrolment and data collection were over the Internet. 405 participants who were 18 to 75 years old and had insomnia completed a two week diary-keeping run-in period without treatment and were randomised and mailed valerian or placebo tablets for two weeks. All participants and investigators were blind to treatment until after the analysis was completed.

**Findings:**

For the primary outcome of a minimally important improvement in self-reported sleep quality (≥0.5 units on a 7 point scale), the difference between the valerian group (29%) and the placebo group (21%) was not statistically significant (difference 7.5%; 95% CI-0.9 to 15.9; p = 0.08). On the global self-assessment question at the end of the treatment period 5.5% (95% CI 0.2 to 10.8) more participants in the valerian group perceived their sleep as better or much better (p = 0.04). There were similar trends favouring the valerian group for night awakenings (difference = 6.0%, 95% CI-0.5 to 12.5) and sleep duration (difference = 7.5%, 95% CI-1.0 to 16.1). There were no serious adverse events and no important or statistically significant differences in minor adverse events.

**Interpretation:**

Based on this and previous studies, valerian appears to be safe, but with modest beneficial effects at most on insomnia compared to placebo. The combined use of television and the Internet in randomised trials offers opportunities to answer questions about the effects of health care interventions and to improve public understanding and use of randomised trials.

**Trial Registration:**

Controlled-Trials.com ISRCTN72748991

## Introduction

This trial of the effectiveness of valerian for insomnia was conducted as part of a collaboration between the Norwegian Knowledge Centre for the Health Services and the Norwegian Broadcasting Corporation (NRK) television program, “Puls”. Puls is a weekly, nationally broadcast program about health. The study was used as an example of a randomised trial in explaining to the television audience some of the principles related to such studies. To our knowledge this is the second trial conducted with this objective [1] and one of a few clinical trials conducted entirely via the Internet [2].

Insomnia is defined by a repeated difficulty with sleep initiation, duration, consolidation, or quality that occurs despite adequate time and opportunity for sleep and results in some form of daytime impairment [3]. More than 50 epidemiological studies have shown that one third of people in a variety of general populations have insomnia symptoms and that 9% to 21% have insomnia with serious daytime consequences, such as fatigue, diminished energy and difficulty concentrating [4]. In Norway, 12% of adults have been found to suffer from insomnia based on standard criteria (DSM-IV) [5]. Insomnia is often co-morbid with underlying psychiatric and medical conditions, and these conditions should be evaluated and treated as a first measure. In this study we excluded people who reported symptoms of secondary insomnia.

A wide range of approaches are used to treat insomnia. The most common is prescription of hypnotics, such as benzodiazepines or newer compounds like zolpidem, zopiclone and eszopiclone. Of non-pharmacological interventions for insomnia cognitive behaviour therapy (CBT) seems to be the best documented [6].

Due to adverse effects [7] and the fact that long-term use of hypnotics normally not is recommended, and the limited availability of CBT, many insomniacs resort to non-prescribed remedies, often based upon herbs. Valerian, in particular, is widely promoted and available without prescription in Norway and elsewhere for treating insomnia [8]–[10].

The root of valerian, a perennial herb native to North America, Asia, and Europe, is believed to have sedative and hypnotic properties. Multiple preparations are available, and the herb is commonly combined with other herbal medications. Valerian (*Valeriana officinalis*) has been suggested for several other uses, including anxiety, depression, menopausal symptoms and stress, but there is limited research documenting its effects for these uses. Valerian is among the eight most widely used herbal supplements [10].

In this trial we tested valerian as a remedy for insomnia. Valerian has long been advocated and used for promoting sleep. During the last 20 years a number of clinical trials have been conducted. Systematic reviews have found inconsistent results and wide variation in the design of the trials [8], [9], [11], [12]. Ten randomised trials of valerian in adults published after 2000 are included in the Cochrane Central Register of Controlled Trials (2007, Issue 1) and MEDLINE (searched using PubMed on 4 April 2007 with ‘valerian’ and publication type ‘randomized controlled trial’) [2], [13]–[21]. All of these studies were included in one or both of the most recently published systematic reviews of valerian [11], [12].

We therefore sought to rigorously evaluate the effects of valerian, the most commonly used herbal product to induce sleep [22] in people with primary insomnia.

## Methods

The protocol for this trial and supporting CONSORT checklist are available as supporting information; see Checklist S1 and Protocol S1. The primary objective of this trial was to evaluate whether valerian improves sleep quality compared with placebo for people with primary insomnia. The secondary objectives were to evaluate valerian's effects on latency to sleep onset, number of night awakenings, total sleep time, daytime energy level, and global self-assessed improvement.

### Inclusion and exclusion criteria

Participants had to be 18 to 75 years old and have suffered from insomnia for more than one month, a Pittsburgh Sleep Quality Index (PSQI) score of >5 [23], Internet access, an email address, and to have completed a sleep diary for at least 10 days in the trial run-in period.

We excluded people with any of the following conditions: use of hypnotics by prescription, depression, alcohol or drug abuse, psychotherapy within the past six months, pregnant or lactating women or women of childbearing potential who did not use oral contraceptives or an intrauterine device, shift workers, a history of hypersensitivity to valerian or its constituents, or current participation in another trial using an investigational compound. We also excluded people who answered ‘usually’ or ‘always’ to the following questions [24]: During the past four weeks, how often

- Did you hold your breath, have breathing pauses, or stop breathing in your sleep?
- Did you snore loudly?
- Did you have restless or “crawling” feelings in your legs at night that went away if you moved your legs?
- Did you have repeated rhythmic leg jerks or leg twitches during your sleep?
- Did you have nightmares, or did you scream, walk, punch, or kick in your sleep?
- Did any of the following disturb you in your sleep: Pain? Other physical symptoms? Medications?

### Recruitment and withdrawals

Information about the study was broadcast nationally in Norway three times between 29 January and 19 February 2007. Viewers interested in participation were invited to visit the web pages of the study to enrol (http://sovnstudien.forskningspuls.no/).

The initial screening of potential participants was conducted online and was automated. Potential participants could contact the study secretariat by email or telephone if they had questions or wanted to discuss the study in more detail. We informed potential participants that we were not providing health care and that they would need to contact their own physician if they became ill during the trial. A study physician was available by telephone throughout the trial to answer questions and provide advice if needed.

We also informed participants that they had the right to withdraw from the study at any time. However, we encouraged them, once they were randomised, to complete the sleep diary for two weeks after they received the study tablets. Participants who withdrew from the study were asked to provide their reasons for withdrawing. Regardless of whether participants completed the sleep diary, we sent up to three email reminders and telephoned participants to obtain their self-assessments of global improvement when they took tablets compared to the two weeks without tablets.

### Randomisation

We assigned an identification number to potential participants who met the inclusion criteria and consented to participate in the trial. After completing the sleep diary for 10 days participants were allocated to valerian or placebo according to a pre-determined randomisation scheme, using a computerised procedure. The corresponding numbers had been printed on the boxes containing the study tablets.

A pharmacy kept the randomisation list and sent the study tablets (either valerian or placebo) as a registered letter, requiring participants to confirm receipt by signature. Participants needed to enter the randomisation code on the tablet box in the sleep diary to verify receipt of the package.

### Intervention

The study treatment was coated tablets containing 200 mg extract per tablet (Valerina Forte®). The manufacturer (Cederroth International AB) stored the placebo and valerian tablets together in a sealed room before putting them into blister packages and shipping the tablets in boxes to the pharmacy, so that the placebo and valerian tablets, which were identical in appearance and taste, would smell the same.

Participants received a box with 60 tablets and instructions to swallow three tablets every night about one hour before going to bed for 14 days. The optimum dose of valerian is unknown [9]. The European Medicines Agency (EMEA) final proposal for Core Data for *Valerianae radix* (http://www.emea.eu.int/pdfs/human/hmpc/001499en.pdf) recommends a single dose of two to three grams one half to one hour before bedtime with an earlier dose during the evening if necessary. Three tablets of Valerina Forte, correspond to 3600 mg *Valeriana officinalis*. This is slightly more than the dosage recommended by EMEA, but less than the maximum dose recommended by the manufacturer (4 tablets) and far below the maximum recommended daily dose of nine grams.

In previous randomised trials using repeated doses, the amount of extract taken per day has ranged from 450 to 1215 mg [9]. It was found in N-of-1 trials that 450 mg (equivalent to 2 grams of dry root and rhizome) was not effective, perhaps due to it being a relatively small dose [16]. We therefore tested a larger dose to ensure the best possibility of observing an effect and because there is not evidence of an increased risk of side effects with the higher dose.

We asked participants to avoid using other medication for insomnia and to continue using their usual self-help strategies for their sleeping difficulties. Upon completion of the study we asked them to return the remaining tablets and the box to the pharmacy using a pre-paid, return-addressed envelope.

### Data collection and outcome measures

**Figure 1:**
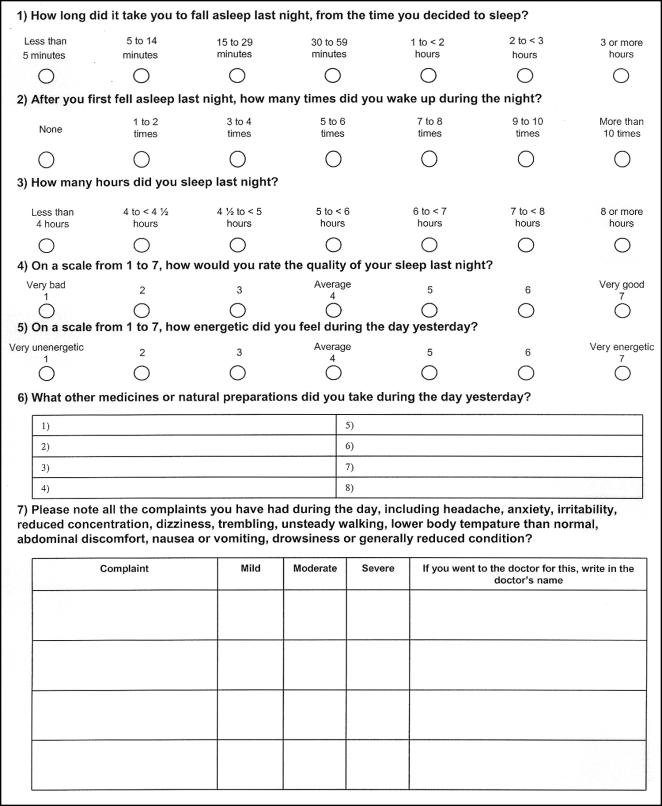
Sleep diary. This is a translation of the diary, which was in Norwegian. The electronic diary included online help, a calendar indicating the number of days completed and remaining, options for viewing graphs of each of the five outcome variables, space for personal notes, and automated checks to ensure that the diary was completed each day and checked for correctness before being submitted. Changes could not be made after the data were submitted. Participants could print out paper versions of the sleep diary and enter the data electronically later if they chose to do so.

Potential participants who met the inclusion criteria and consented to participate were automatically sent an email. They needed to respond to this to activate their user name and password and access their sleep diary. Participants completed a structured online sleep diary every day during the study (figure 1), 14 days prior to starting to take the tablets and 14 days after. Data entered into the diary were automatically stored in a database. Participants were asked to check that the information they entered was correct before saving it. An automated email reminder was sent to participants who had not completed their sleep diary by 1 p.m. each day.

Demographic data were collected from participants at the beginning of the study, including age, sex and education. The PSQI, which was used to screen potential participants for insomnia, includes 19 self-reported items and uses an algorithm to derive a global score from components of sleep quality, sleep latency, sleep duration, habitual sleep efficiency, sleep disturbance, use of sleeping medication and daytime functioning [23]. Scores range from zero to 21. Higher scores indicate more severe sleep problems. A score of above 5 indicates a “poor” sleeper.

The sleep diary (figure 1) included five outcome measures that were recorded daily: sleep onset latency, number of night awakenings, sleep duration, sleep quality, and energy level the following day.

These outcome measures have been used and cited in the literature as being important measures of sleep [9], [16], [25]–[28]. The response options that we used were modified from those used by Coxeter and colleagues [16]. We used seven categories for all five questions for consistency and to facilitate interpretation relative to what has been found to be a clinically important mean difference on 7-point scales [29]. We added an additional category at the upper end of sleep latency and we used half-hour intervals for total sleep time under five hours to detect what are likely to be meaningful differences for these two outcomes, based on clinical experience.

In addition the sleep diary included questions about adverse events, concomitant medication, and the number of tablets taken. At the end of the study participants were asked to assess their sleeping problems during the two weeks when they took tablets compared to the two week run-in period on a 7-point scale ranging from much worse to much better. They were also asked how many tablets they had left and were asked whether they thought the tablets were valerian, placebo or they did not know.

### Adverse events

Adverse events were defined as any undesirable experience occurring to a participant during the study, whether or not considered related to the study medication. Serious adverse events were defined as an adverse experience that was fatal, life-threatening, disabling or which resulted in-patient hospitalisation or prolongation of hospitalisation. Unexpected adverse events were defined as an experience not previously reported in the product information sheet or similar documents.

Participants were asked to record all complaints that they had each day; to indicate whether each of these was mild, moderate or severe; and to note if a physician was seen because of the complaint. We monitored the sleep diary for severe side-effects in order to obtain information about potential serious adverse events if needed. When participants registered a complaint in the sleep diary as “severe”, a message was automatically sent to the secretariat and we determined whether these were serious adverse events and whether we needed to contact the participant or physician to obtain additional information.

### Analysis

The primary outcome was the proportion of participants in each group with an improvement in self-reported sleep quality of ≥0.5 units between the average score for the two weeks before and two weeks during treatment. This was based on what we assumed likely to be a clinically important difference, based on findings from other 7-point scales [29]. The two proportions were compared using a chi-square test.

Secondary analyses included comparisons of the proportion of participants in each group with an improvement of ≥0.5 units between the average score for the two weeks before and two weeks during treatment for the other four outcome measures: sleep onset latency, the number of awakenings, sleep duration, and daytime energy level. These were also compared using chi-square tests. In addition, we compared the mean change for each of the five outcome measures using two-sample t-tests. For the participants' global self-assessments of change in their sleeping problems we compared the proportion of participants that reported an improvement using two cut off points-better or much better, and any improvement-using chi-square tests.

We conducted an exploratory analysis where we compared the proportion of participants in each group with any improvement in mean difference (i.e. >0) between the average score for the two weeks before and two weeks during treatment for each of the five variables using chi-square tests. We also conducted an exploratory analysis using repeated measures analysis of variance for all five outcome measures in order to reveal any difference in the profile of the five endpoints during the intervention period taking into account the run-in period and we evaluated whether there were differences in effect over time.

Safety analyses included tabulation of type and frequency of all adverse events. We compared the proportion of participants in each group recording one or more adverse events during the intervention compared to the run-in period using McNemar's test and chi-square tests for the between group comparisons.

All analyses were done on an “intention-to-treat” basis; i.e. all participants who reported receiving study drugs and taking them at least once were included. All p-values and confidence intervals are reported without adjustments for multiple comparisons, and have been interpreted in light of the number of comparisons that were made. The analyses were done using SAS (Version 9.1.3. SAS Institute Inc., Cary, NC, USA).

The randomisation code was broken in two steps. The participants were identified as belonging to group “A” or “B” without revealing which was the valerian and placebo group. The “A” and “B” code was not broken until the primary and secondary statistical analyses had been completed and reviewed by the investigators.

### Sample size calculations

We estimated the number of participants needed to reject the null hypothesis (no difference between valerian and placebo) for the primary analysis for proportions from 0.15 to 0.40 in the placebo group and absolute improvements of 10 to 25% based on Pearson Chi-square 2-sided tests for two proportions, with a significance level of 0.05 and 80% power. We did not allow for drop-outs since all participants who completed the sleep diary for at least one day were included in all of the analyses except for global self-assessed improvement. The calculations were performed with SAS, PROC POWER. The estimated sample sizes ranged from 58 to 388 per group, depending on the proportion in the placebo group and the minimum difference considered to be important. We intended to include 250–275 participants in each group in order to be able to reject the null hypothesis if the difference in proportions was between 10 and 15%.

### Ethical considerations

The protocol, participant information and informed consent form were approved by the Regional Committee for Medical Research Ethics–Southern Norway, the Norwegian Medicines Agency and the Norwegian Social Science Data Services. The trial was registered and assigned an International Standard Randomised Controlled Trial Number (ISRCTN72748991) by Current Controlled Trials (www.controlled-trials.com) prior to commencing recruitment. The protocol is publicly available (http://www.kunnskapssenteret.no/filer/sovnstudien_protokoll_3_1_2_2007_01_3.pdf).

Study participants were informed that they were free to withdraw from the study at any time and that there was no risk associated with discontinuing taking the tablets. Information about the study was provided to all potential participants on the web pages. The participants could take as much time as they needed to read and understand the information before consenting to participate (electronically). They had the opportunity to contact the investigators by phone or email and ask questions before deciding whether they wanted to participate in the study. In order to participate, potential participants had to answer several questions to ensure that they understood the study procedures and potential harms and benefits before they verified their consent to participate online.

Because the study was used to educate a television audience regarding principles of randomised trials, journalists wanted to interview and follow some of the participants in the study. The participants were asked upon inclusion whether they would be interested in being contacted by a journalist. It was emphasized that this was not a prerequisite for participation and that only two to four participants would be contacted.

We kept a separate log of participants' codes and names. This database also included the electronic confirmation of each person's consent to take part in the study. The sleep diary was kept in a secure zone. Each participant was able to access her or his own sleep diary and no one else's. The investigators had access only to anonymous data in tables.

## Results

**Figure 2:**
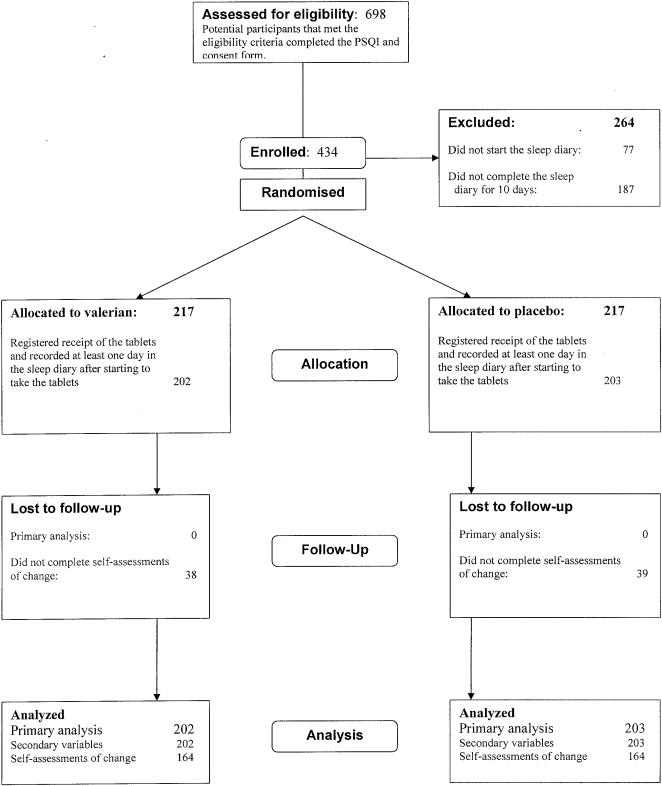
Flowchart

Information about the study was first broadcast on Puls on 29 January 2007. 698 eligible participants had completed the informed consent by the end of the recruitment period, 5 March 2007 (figure 2). Of those 434 were eligible for randomisation after completing the sleep diary for 10 days and were randomised. 405 people (202 in the valerian group and 203 in the placebo group) subsequently filled in at least one day of the sleep diary after starting to take the tablets and were included in the analyses. 328 participants completed the final global self-assessments of change (164 in each group). All other analyses included all 405 people who registered receipt of the tablets and took them for at least one day.

The two groups were similar with the exception of age (table 1). The median score on the PSQI was 11 (range 7 to 18). The median sleep duration for the month prior to entering the study was six hours and 75% reported having very bad sleep quality.

**Table 1:** Characteristics of the participants

	Valerian	Placebo
	(N = 202)	(N = 203)
Sex (% men)	38	40
Age		
Mean (standard deviation)	45.7 (13.8)	41.8 (12.9)
Median (range)	49.0 (17–74)	42.0 (18–73)
Education		
Primary school (%)	7	5
Secondary school (%)	24	28
College or university (%)	69	67
Pittsburgh Sleep Quality Index (PSQI)		
Mean (standard deviation)	11.6 ( 2.7)	11.5 ( 2.6)
Median (range)	11.0 ( 7–18)	11.0 ( 7–18)
>60 minutes to fall asleep (%)	61	66
Minutes of sleep per night (N = 201 and 203)		
Mean (standard deviation)	312 (62)	318 (64)
Median (range)	300 (120–570)	300 ( 45–600)
Very bad sleep quality (%)	72	75
Regular use of medication–not for insomnia (%)	50	54

* PSQI scores range from 0 to 21. Higher scores indicate more sever sleep problems. A score of >5 indicates a “poor” sleeper.

The two groups also had similar results during the two-week diary-keeping run-in period prior to randomisation (table 2). The median quality of sleep for the 14 days prior to the intervention was 3.9, corresponding to “average” on a scale from 1 (very bad) to 7 (very good). The median on the scale for time to fall asleep was 3.5, corresponding to approximately 30 minutes. The median on the night awakenings scale was 2.4, corresponding to 2 or 3 times. The median on the sleep duration scale was 4.1, corresponding to 5 to 6 hours, and the median on the energy level scale was 3.9, corresponding to “average” on a scale from 1 (very unenergetic) to 7 (very energetic).

**Table 2:** Run-in period results

	Valerian (N = 202)	Placebo (N = 203)
Sleep quality (1 = very bad to 7 = very good)		
Mean (standard deviation)	3.89 (0.83)	3.91 (0.75)
Median (range)	3.93 (1.00–6.36)	3.93 (1.29–6.14)
asleep onset latency (1 = <5 minutes to 7 = ≥3 hours)		
Mean (standard deviation)	3.61 (1.13)	3.47 (1.04)
Median (range)	3.56 (1.00–7.00)	3.36 (1.14–6.93)
Night awakenings (1 = none to 7 = >10 times)		
Mean (standard deviation)	2.53 (0.93)	2.40 (0.80)
Median (range)	2.43 (1.00–7.00)	2.29 (1.00–6.79)
Sleep duration (1 = <4 hours to 7 = ≥8 hours)		
Mean (standard deviation)	3.98 (1.04)	4.08 (0.98)
Median (range)	4.00 (1.00–6.79)	4.15 (1.14–6.21)
Energy level next day (1 = very unenergetic to 7 = very energetic)		
Mean (standard deviation)	3.91 (0.80)	3.83 (0.68)
Median (range)	3.88 (1.71–6.50)	3.86 (1.79–6.14)

On average participants took the tablets for 13 days (median 14) in both groups during the intervention period (mean 12.9 (SD 3.0) for the valerian group and 13.0 (SD 3.1) for the placebo group. 89% of the valerian group and 90% of the placebo group took the tablets for 14 or more days. Fewer than 5% in both groups took the tablets for four or fewer days. 57 participants reported a reason for not taking the tablets for the full 14 days. The most common reasons were not wanting to bother anymore with the sleep diary (14), travel or problems with access to the Internet (11), influenza or a cold (5), no improvement or complaints attributed to the tablets (4), and improved sleep (4).

### Effectiveness of valerian

28.7% of the valerian group and 21.2% of the placebo group had a minimally important improvement (≥0.5) on the sleep quality scale (table 3), the primary outcome measure. This difference was not statistically significant (difference 7.5%; 95% CI-0.9 to 15.9; p = 0.08). There were similar trends favouring the valerian group for night awakenings (difference = 6.0%, 95% CI -0.5 to 12.5) and sleep duration (difference = 7.5%, 95% CI-1.0 to 16.1). There was little difference in the average change in scores for all five outcome measures (0.01 to 0.10 in favour of varerian) and only the difference for night awakenings was statistically significant (mean difference = 0.09, 95% CI 0.01 to 0.18). The differences were small and not statistically significant for any of the comparisons of the proportions of participants with any improvement (−7.6% in favour of placebo to 5.2% in favour of valerian).

**Table 3:** Effectiveness results

	Valerian (N = 202)	Placebo (N = 203)	Difference (95% CI)	P-value
Sleep quality (1 = very bad to 7 = very good)				
- Improvement ≥0.5 (%)	28.7	21.2	7.5 ( −0.9 − 15.9)	0.08
- Any improvement (%)	62.9	58.1	4.7 ( −4.8 − 14.3)	0.33
- Average improvement– Mean (standard deviation)	0.23 (0.65)	0.15 (0.57)	0.08 (−0.04 − 0.20)	0.21
– Median (range)]	0.15 (−1.70 − 2.46)	0.07 (−1.72 − 2.14)		
Sleep onset latency (1 = <5 minutes to 7 = >3 hours)*				
- Improvement≥0.5 (%)	24.3	21.2	3.1 ( −5.1 − 11.2)	0.46
- Any improvement (%)	58.9	66.5	−7.6 ( −17.0 − 1.8)	0.11
- Average improvement – Mean (standard deviation)	0.15 (0.63)	0.14 (0.52)	0.01 (−0.11 − 0.12)	0.89
– Median (range)	0.15 (−3.93 − 1.93)	0.15 (−1.35 − 1.52)		
Night awakenings (1 = none to 7 = >10 times)*				
- Improvement≥0.5 (%)	15.8	9.9	6.0 ( −0.5 − 12.5)	0.07
- Any improvement (%)	60.4	55.2	5.2 ( −4.4 − 14.8)	0.29
- Average improvement – Mean (standard deviation)	0.13 (0.42)	0.03 (0.41)	0.09 (0.01 − 0.18)	0.02
– Median (range)	0.07 (−1.11 − 1.33)	0.05 (−1.64 − 1.19)		
Sleep duration (1 = <4 hours to 7 = >8 hours)				
- Improvement ≥0.5 (%)	30.2	22.7	7.5 ( −1.0 − 16.1)	0.09
- Any improvement (%)	67.8	65.5	2.3 ( −6.9 − 11.5)	0.62
- Average improvement – Mean (standard deviation)	0.28 (0.64)	0.17 (0.49)	0.10 (−0.01 − 0.21)	0.07
– Median (range)	0.23 (−4.07 − 2.14)	0.16 (−1.04 − 2.07)		
Energy level next day (1 = very unenergetic to 7 = very energetic)				
- Improvement ≥0.5 (%)	26.2	23.6	2.6 ( −5.8 − 11.0)	0.55
- Any improvement (%)	60.4	56.7	3.7 ( −5.8 − 13.3)	0.44
- Average improvement – Mean (standard deviation)	0.17 (0.61)	0.14 (0.62)	0.03 (−0.09 − 0.15)	0.60
– Median (range)	0.14 (−1.33 − 2.00)	0.10 (−2.01 − 1.93)		
Global self-assessment of change	(N = 164)	(N = 164)		
- Much better or better (%)	9.1	3.7	5.5 ( 0.2 − 10.8)	0.04
- Any improvement (%)	26.8	21.3	5.5 ( −3.7 − 14.7)	0.25

* Although higher scores were worse for these outcomes, the results are reported in terms of improvements, consistent with the other outcomes.

5.5% (95% CI 0.2 to 10.8) more participants in the valerian group compared to the placebo group perceived their sleep as better or much better on the global self-assessment question at the end of the treatment period (p = 0.04) (table 3). The difference between the two groups for any self-assessed improvement was the same (5.5%, 95% CI-3.7 to 14.7, p = 0.25), as there was no difference in the proportions in the two groups that indicated they were a little better. Chi-square tests showed that the participants, own perception of improvement on the global self-assessment did not correspond to improvements on the five outcome measures recorded in the sleep-diary (p<0.005).

There were no statistically significant effects of treatment for any of the five outcome measures in the repeated measures analysis of variance (p = 0.06 for night awakenings to 0.89 for sleep onset latency; p = 0.31 for sleep quality). The mean differences varied from day to day for all five variables from 0.01 to 0.97. There was no apparent pattern to the differences and none of the interactions between treatment and time were statistically significant (p = 0.11 to 0.82).

### Adverse events

There were no statistically significant differences (p≥0.20) between the two groups for any type of complaint (mild, moderate or severe) or the number of days with complaints during the run-in or intervention periods (table 4). Although 24% of participants in the run-in period and 17% in the treatment reported “severe” complaints, none of the participants experienced a serious adverse event during the study period. The majority of complaints could be consequences of insomnia (drowsiness, tiredness, reduced concentration, irritability, uneasiness, headache, dizziness, trembling). Other complaints were unlikely to be related to valerian (cold and influenza symptoms, eye infection, back and joint pains).

**Table 4:** Adverse events

	Valerian (N = 202)	Placebo (N = 203)	Difference	P-value
Mild complaints*				
Run-in period (%)	74	68	5.8	0.20
Treatment period (%)	50	51	−1.7	0.73
Moderate complaints*				
Run-in period (%)	60	65	−4.6	0.34
Treatment period (%)	48	42	5.1	0.30
Severe complaints*				
Run-in period (%)	25	24	0.6	0.89
Treatment period (%)	18	16	2.1	0.58
Days with complaints				
Percent	24	23	1.0	0.73
Average (SD)	3.0 (4.4)	2.9 (4.1)	0.1	0.70

* Self-reported severity

82% of the participants reported complaints during the run-in period and 62% during the treatment periods. The difference in the proportion of participants within each treatment group reporting complaints during the run-in period compared to the intervention period was statistically significant (McNemar's p<0.001).

### Participants' guesses about which tablets they received

There were 8% more people in the valerian group who believed that they had received valerian at the end of the study compared to the placebo group (17 versus 9%), 17% fewer who believed they had received placebo (51 versus 68%), and 9% more who responded that they did not know (32 versus 23%). These differences were statistically significant (p = 0.004).

## Discussion

Our trial was nearly twice as large as any previously reported trial of valerian. Although we did not achieve our pre-trial intended sample size, we are able to rule out the possibility that valerian has a large beneficial effect. The best estimate of the effect of valerian on sleep quality, our main outcome measure, suggests that roughly 13 people with primary insomnia would need to be treated for one additional person to experience a noticeable improvement (NNT). However, this result was not statistically significant and we cannot rule out either that valerian is actually worse than placebo, or that the NNT could be as small as 6, although this appears unlikely. There was a trend towards similar effects for night awakenings and sleep duration, and a marginally statistically significant effect on global self-assessed improvement of much better or better (NNT = 18, 95% CI 9 to 500). There were no other statistically significant effects. We did not adjust for multiple comparisons and given the number of comparisons made, it is possible that all of the observed differences could simply reflect the play of chance.

There is a low risk of the results being biased. The randomisation resulted in comparable groups and only 29 people who were randomised following the run-in period (7%) did not take the tablets and were not included in the primary analysis or most of the secondary analyses. Equal proportions of participants (19%) in both groups did not answer the global self-assessment of change question and were not included in that analysis. Although more people in the valerian group than the placebo group thought that they had received valerian (17 versus 9%), this most likely is due to people who improved assuming that they were in the valerian group and people who did not improve assuming they were in the placebo group [30]. Of the 43 participants who thought they received valerian 25.6% reported being better or much better compared to 0.5% of the 195 participants who thought they received placebo (p<0.001). There were similar differences across the other outcomes.

Recruitment for this study was completed in one month, data collection was completed one month after that and it was possible to analyse the data within days, since all data were reported online by the participants and entered directly into a database. The time required for recruitment and follow-up depends, of course, on the condition and treatment being studied. However, this trial demonstrates that the combination of television and the Internet can be effective and efficient tools for conducting trials.

### What was already known and what does this study add

Systematic reviews have concluded that there is uncertainty about the effectiveness of valerian [8], [9], [11], [12]. A meta-analysis published in 2005 that included three randomised trials with a total of 99 participants that reported sleep quality [11] a statistically non-significant improvement with valerian (standardised mean difference 1.38, 95% CI-0.49 to 3.25) with substantial heterogeneity among the trials (I^2^ = 90.3%). A more recent review published in 2006 that included 16 randomised trials that reported sleep quality found that most studies had significant methodological problems [12]. A meta-analysis of six studies with a total of 847 participants that reported sleep quality improvement as a dichotomous outcome found a statistically significant improvement with valerian (relative risk 1.8, 95% CI 1.2 to 2.9). However there was substantial heterogeneity of the results, inclusion criteria, dose, duration and methodological quality of these studies, as well as evidence of publication bias. Overall, these results suggest that if valerian has any effect on sleep quality, it is small.

A meta-analysis of three trials did not find an important effect on sleep onset latency (weighted mean difference −1.3 minutes, 95% CI-21.4 to 18.9) and also found substantial heterogeneity for this outcome (I^2^ = 77.6%) [11]. A more recent trial [22] not included in that meta-analysis reported a small difference in sleep onset latency in favour of placebo, which was not statistically significant (8.3 minutes, 95% CI-0.3 to 16.8 minutes). We also did not find any statistically significant difference for sleep onset latency (mean improvement = 0.01, 95% CI-0.11 to 0.12).

Only one randomised trial with 19 participants included in that review [11] reported sleep duration. It found a difference of 0.8 minutes (95% CI-50.6 to 52.2), comparable with our finding of a very small difference (mean improvement = 0.10, 95% CI-0.01 to 0.21).

In summary, this randomised trial confirms that valerian is unlikely to reduce the time it takes to fall asleep or daytime energy level. Valerian may have a small effect on sleep quality and global self-assessed improvement, but only a small number of people with insomnia are likely to experience a noticeable improvement attributable to valerian.

### Implications

Valerian appears to be safe, but with very modest beneficial effects on insomnia. The fact that valerian has any effects at all may be a reason to explore how these effects might be enhanced. Unless there are advances in understanding of this kind, further pragmatic trials are unlikely to be worthwhile.

This trial was the first televised clinical trial conducted as part of the “ForskningsPuls” (Research Pulse) project, which involves the television audience in the trial and uses the trial to illustrate principles of evaluating health care interventions. The aim of this project is to enhance public understanding and use of randomised trials to inform decisions about healthcare. So far as we know, it is the second trial undertaken with this in mind [11], and it is one of relatively few trials conducted entirely on the Internet [22]. Although there are limitations to what it is feasible to evaluate in this way, we believe that this approach offers important opportunities both to answer questions about the effects of health care interventions and to improve public understanding and use of randomised trials.

## Supporting Information

Protocol S1 Trial protocol
(0.16 MB PDF)

Checklist S1 CONSORT checklist
(0.06 MB DOC)
