# Supplementary material for: A Televised, Web-Based Randomised Trial of an Herbal Remedy (Valerian) for Insomnia
Source: PLoS One. 2007 Oct 17;2(10):e1040. doi: 10.1371/journal.pone.0001040 (PMC2002515; doi:10.1371/journal.pone.0001040)
Supplement: Protocol S1 — Trial protocol (0.16 MB PDF) [file pone.0001040.s001.pdf]

CLINICAL STUDY PROTOCOL

**Protocol no.:**	2005001TVT
**Title:**	A Televised, Web-based Randomised Trial of a Herbal Remedy (Valerian) for Insomnia
**Indication:**	Insomnia
**Investigational product:**	Valerian
**Development phase:**	IV

Project Coordination Group:

- Andrew D Oxman*
- Signe Flottorp*
- Cheryl Carling*
- Atle Fretheim*
- Doris Tove Kristoffersen*
- Torbjørn Fosen Wisløff*
- Astrid Dahlgren*
  * Norwegian Knowledge Centre for the Health Services
- Ståle Pallesen
  Department of Psychosocial Science, University of Bergen, Norwegian Competence Center for Sleep Disorders, Haukeland University Hospital
- Bjørn Bjorvatn, University of Bergen
  Section for General Practice, University of Bergen, Norwegian Competence Center for Sleep Disorders, Haukeland University Hospital

Sponsor: Norwegian Knowledge Centre for the Health Services, P.O.Box 7004 St Olavs plass, N-0130 Oslo, Norway. Tel.: +47 23 25 50 00. Fax: +47 23 25 50 10 Email: oxman@online.no

Protocol date and version: 31 January 2007 – version 3.2

ISBN 978-82-8121-116-2

Oxman, Andrew
Clinical study protocol [Protocol no 2005001TVT]
A televised, Web-based Randomised Trial of a herbal remedy (Valerian) for Insomnia
Protokoll mai 2007

TABLE OF CONTENTS

CLINICAL STUDY PROTOCOL 1

TABLE OF CONTENTS 2

PROTOCOL OUTLINE 4

LIST OF ABBREVIATIONS 7

1 INTRODUCTION 8

2 OBJECTIVES 9

3 POPULATION 9

3.1 INCLUSION CRITERIA 9
3.2 EXCLUSION CRITERIA 10
3.3 RECRUITMENT AND SCREENING 10
3.4 PARTICIPANT WITHDRAWAL 10

4 TREATMENT PROCEDURES 11

4.1 ASSIGNMENT TO TREATMENT 11
4.2 ASSESSMENTS AND PROCEDURES 11
4.3 SCHEDULING OF PROCEDURES 14

5 STUDY TREATMENT 15

5.1 STUDY TREATMENT AND COMPARATOR 15
5.2 SUPPLY, PACKAGING, LABELLING, HANDLING AND STORAGE 15
5.3 DOSAGE AND ADMINISTRATION 16
5.4 DURATION OF TREATMENT 16
5.5 CONCOMITANT MEDICATION 16
5.6 STUDY MEDICATION ACCOUNTABILITY 16

6 RESPONSE VARIABLES AND ENDPOINTS 17

6.1 ASSESSMENT OF EFFECTIVENESS 17
6.1.1 Primary effectiveness variable 17
6.1.2 Secondary effectiveness variables 17
6.2 ASSESSMENT OF SAFETY 17
6.3 ADVERSE EVENTS AND SERIOUS ADVERSE EVENTS 17
6.3.1 Definitions of Adverse Events 17

SEVERITY 18

RELATIONSHIP TO STUDY MEDICATION 18

6.3.2 Reporting of adverse events 19

7 STATISTICAL METHODS AND DATA MANAGEMENT 19

7.1 STUDY DESIGN 19
7.2 ESTIMATION OF SAMPLE SIZE 19
7.3 RANDOMISATION 20
7.4 BLINDING AND CODE BREAKING INSTRUCTIONS 20
7.5 STATISTICAL ANALYSIS PLAN 20
7.6 STUDY POPULATIONS 21

INTENTION-TO-TREAT POPULATION 21

7.7 DATA COLLECTION / CASE REPORT FORMS 22

7.8 DATA MANAGEMENT 22

8 REGULATORY AND ADMINISTRATIVE PROCEDURES 22

8.1 ETHICS COMMITTEE 22
8.2 PARTICIPANT INFORMATION / INFORMED CONSENT....22
8.3 PARTICIPANT CONFIDENTIALITY 23
8.4 PARTICIPANT TREATMENT PLAN 23
8.5 GCP....23
8.6 ESSENTIAL DOCUMENTS 23
8.7 RECORD RETENTION 24
8.8 MONITORING / QUALITY CONTROL....24
8.9 QUALITY ASSURANCE 24
8.10 INSURANCE AND LIABILITY 24
8.11 END OF TRIAL....24
8.12 STUDY REPORT 24
8.13 PUBLICATION AND DATA RIGHTS 24

9 REFERENCES 26

10 SIGNATURES....28

11 APPENDICES 29

11.1 STUDY FLOW-CHART 29
11.2 PATIENT INFORMATION/INFORMED CONSENT FORM 30

PROTOCOL OUTLINE

<table>
<tr><td colspan="2">Title</td><td>A Televised, Web-based Randomised Trial of a Herbal Remedy (Valerian) for Insomnia</td></tr>
<tr><td colspan="2">Objectives</td><td>The primary objective is to evaluate whether valerian root (valerian) improves the self-assessed quality of sleep compared with placebo for people with primary insomnia.

The secondary objectives are to evaluate
• Latency to sleep onset
• Number of night awakenings
• Total sleep time
• Energy level in the following day</td></tr>
<tr><td colspan="2">Study design</td><td>Randomised, double-blind, parallel group, placebo controlled trial</td></tr>
<tr><td rowspan="2">Effectiveness variables</td><td>Primary</td><td>Proportion of participants in each group with an improvement in self-assessed quality of sleep of ≥ 0.5 units between the average score for the 2 weeks before and 2 weeks during treatment</td></tr>
<tr><td>Secondary</td><td>o Proportion of participants in each group with an improvement of ≥ 0.5 units between the average score for the 2 weeks before and 2 weeks during treatment for each of the four variables:
▪ sleep latency
▪ number of awakenings
▪ total sleep time
▪ energy level during the day

o Mean changes in the five outcomes listed above.

o Global self-assessment

If a difference is not found between the treatment groups for the primary and secondary variables, the following explorative variables will be analysed.

o Proportion of participants in each group with any improvement in mean difference (i.e. all score changes > 0) between the average score for the 2 weeks before and 2 weeks during treatment for each of the five variables:
▪ quality of sleep
▪ sleep latency
▪ number of awakenings
▪ total sleep time
▪ energy level during the day

o Difference in the profile of the five endpoints during the intervention period taking into account the baseline period, including a potential time effect.</td></tr>
<tr><td colspan="2">Safety variables</td><td>Evaluation of adverse events</td></tr>
<tr><td>Selection criteria</td><td>Inclusion</td><td>1. Aged 18 –75 years, both inclusive
2. Insomnia lasting more than one month
3. PSQI score of > 5
4. Access to Internet and own email address
5. At least 10 days of the sleep diary completed prior to randomisation</td></tr>
</table>

<table>
<tr><td></td><td>Exclusion</td><td>1. Secondary insomnia
2. Use of hypnotics by prescription
3. Depression
4. Alcohol or drug abuse
5. Psychotherapy within the past six months
6. Sleep apnoea, periodic limb movements disorder or restless legs syndrome
7. Pregnant or lactating women or women of childbearing potential who do not use an approved method of contraception (oral contraceptives or IUD (Intrauterine device))
8. Shift workers
9. History of hypersensitivity to valerian or its constituents
10. Participant rating of ‘usually’ or ‘always’ to the following questions in the Global Sleep Assessment Questionnaire:
During the past four weeks, how often:
• Did you hold your breath, have breathing pauses, or stop breathing in your sleep?
• Did you have restless or "crawling" feelings in your legs at night that went away if you moved your legs?
• Did you have repeated rhythmic leg jerks or leg twitches during your sleep?
• Did you have nightmares, or did you scream, walk, punch, or kick in your sleep?
• Did any of the following disturb you in your sleep:
- Pain?
- Other physical symptoms?
- Medications?
• Did you snore loudly?
11. Current participation in another trial using an investigational compound</td></tr>
<tr><td colspan="2">Methods & procedures</td><td>Participants will be recruited by use of television, and the participant registrations and data collection will be by use of internet.

Participants who fulfil all of the inclusion criteria and none of the exclusion criteria will be included in the study (Visit 1) and undergo a 14 days non-treatment period. A sleep diary will be completed electronically every day during the whole study. At Visit 2 (Day 10) the selection criteria are once more confirmed, and the participant’s ability to complete the sleep diary is assessed. If the participant still fulfils all selection criteria and has completed the sleep diary every day, he/she can be randomised to study treatment.

The participant will start taking the study treatment at Visit 3 (Day 15).

The treatment period will be 14 days upon the participant will answer questions related to overall assessment of treatment (Visit 4).</td></tr>
<tr><td rowspan="2">Treatment (dose, route, duration)</td><td>Test</td><td>Coated Valerian Forte tablets 200 mg extract per tablet, corresponding to 1200 mg Valeriana officinalis
3 tablets to be taken every night for 14 days</td></tr>
<tr><td>Comparator(s)</td><td>Placebo tablets</td></tr>
<tr><td>Statistics</td><td>Sample size</td><td>Sample size calculations are based on Pearson Chi-square 2-sided test for two proportions, with a significance level of 0.05; power set to 80%
By including about 250-275 participants in each group we would be able to reject the null hypothesis if the difference in proportions is a about 10-15%.</td></tr>
</table>

	Methods	Demographic data will be summarised by treatment group using means, minimums, medians, maximums and standard deviations for normally distributed variables and frequency counts and percents for categorical variables. This information will be used to describe the participants and compare the two groups with respect to these variables.<br><br>For each outcome (quality of sleep, latency to sleep onset, number of awakenings, total sleep time, energy level the previous day) we will report the proportion of participants in each group with an improvement and the risk ratio with 95% confidence intervals. In addition, the mean values will be calculated for each participant for both the baseline period and the intervention period as well as the change in mean score per participant for each or these five outcomes. The changes will be summarized by means, minimums, medians, maximums, standard deviations and the associated 95% confidence intervals.<br><br>Safety analyses will include tabulation of type and frequency of all adverse events. Any serious adverse events will be reported with comprehensive narratives.<br><br>*Primary analysis*<br>The primary analysis will compare the proportion of participants reporting an improvement in mean score of 0.5 for the quality of sleep, by a chi-square test. We will report the difference in proportion and the associated 95% confidence interval.<br><br>*Secondary analyses will comprise*<br>• Comparison of the proportion of participants reporting an improvement in mean score of 0.5 for the other four outcomes (sleep latency, number of awakenings, total sleep time, and energy level during the day) by chi-square tests<br>• Comparison of the proportion of participants with any improvement for each of the five outcomes by chi-square tests<br>• Comparison of the proportion of participants reporting improvement on the global self-assessment using two cut off points (better or much better, and any improvement) by chi-square tests<br>• Comparison of the mean changes in the five outcomes by two-sample t-tests.<br>• Comparison of the proportion of participants in each group recording one or more adverse events during the intervention compared to the run-in period by MacNamars test and a chi-square test for the between group comparison<br>• An explorative analysis using repeated measures analysis of variance techniques in order to reveal any difference in the profile of the five endpoints during the intervention period taking into account the baseline period. This will also comprise a possibility to evaluate a time effect, e.g. to evaluate whether the effect is different after first day of treatment compared to a later day of treatment.<br><br>The confidence intervals will be reported without adjustments for multiple comparisons, and will be interpreted in the light of number of comparisons that are made

LIST OF ABBREVIATIONS

AE	Adverse Event
CRF	Case Report Form
EMEA	The European Medicines Agency
GCP	Good Clinical Practice
ICH	International Conference of Harmonisation
ITT	Intention-to-treat
OTC	Over-the-counter
PSQI	Pittsburgh Sleep Quality Index
SAE	Serious Adverse Event
SDV	Source Data Verification

1 INTRODUCTION

Insomnia is a common problem of insufficient or poor quality sleep, with adverse daytime consequences. The symptoms of insomnia include difficulty falling asleep (long-sleep latency), difficulty staying asleep (excessive or prolonged awakenings), or feeling non-restored from sleep. Insomnia can be a primary disorder or might be a consequence of a psychiatric, medical or other sleep disorders. It can be transient or chronic. Persistent untreated insomnia is a strong risk factor for major depression. More than 50 epidemiological studies have shown that one third of people in a variety of general populations have insomnia symptoms and that 9% to 21% have insomnia with serious daytime consequences, such as bodily fatigue, diminished energy, difficulty concentrating, memory impairment, low motivation, loss of productivity, irritability, interpersonal difficulties (with family, friends, co-workers), increased worrying, anxiety, and depression.[1]

The reported prevalence of insomnia depends on the methods and definitions used in the epidemiological studies. A wide range has been reported for prevalence, from 5 to 35% of people, depending on the descriptors used, severity, duration, age group, and sampling bias. The importance of distinguishing between insomnia symptoms and genuine dissatisfaction with sleep is underscored by the findings of one epidemiological study,[2] which showed that 36% of people reported insomnia but only one in four of these described sleep dissatisfaction. People who have dissatisfaction with sleep have characteristics most usually associated with clinically important insomnia. In another study [3], researchers used somewhat more stringent criteria (including a requirement of daytime consequences), which still yielded a prevalence for severe insomnia of 10–15%. Rates of insomnia with associated daytime dysfunction in attendees of general medical practices range from 10% to as high as 34%.[4, 5, 6, 7] For high quality studies investigating general populations, the prevalence of chronic insomnia ranged from 5-45%, and the median was 17.6%.[8] In Norway 12% of adults have been found to suffer from insomnia based on standard criteria (DSM-IV).[9]

Insomnia is often secondary to underlying psychiatric and medical conditions, and these conditions should be evaluated and treated as a first measure. In this study we will focus on adults with primary insomnia, that is sleep problems not associated with other health problems.

A wide range of remedies is used to treat insomnia. These include establishing daily habits to promote sleep, such as exercise, and minimizing habits that interfere with sleep, such as drinking coffee in the evening. Various types of training can be used to help people with insomnia to change habits such as these (“sleep hygiene training”), using written information, audio recordings, Internet-based information, or personal advice. Relaxation training and stimulus control can also be used. Other remedies include cognitive behavioural therapy, relaxation techniques, meditation, yoga, and light therapy.[1, 4] Prescription drugs, especially benzodiazepines, are also widely used, although these drugs have a number of adverse effects.[10] A wide range of herbs and drugs are used to treat insomnia. Valerian, in particular, is widely promoted and available without prescription in Norway and elsewhere for treating insomnia.[11, 12]

People with insomnia use the root of valerian, a perennial herb native to North America, Asia, and Europe, most commonly for its sedative and hypnotic properties. Multiple preparations are available, and the herb is commonly combined with other herbal medications. Valerian (*Valeriana officinalis*) has been suggested for a wide range of other uses, including anxiety, depression, menopausal symptoms and stress, but there is limited research documenting its effects for these other uses.

We propose to test valerian, a remedy for insomnia. Valerian has long been advocated and used for promoting sleep but until quite recently evidence was anecdotal, rather than based on research. During the last 20 years a number of clinical trials have been conducted. A systematic review of nine trials published between 1984 and 1996 found inconsistent results and wide variation in the design of the trials.[12] Thus, the evidence for valerian as a treatment for insomnia was inconclusive at that time. A more recent review found 18 experimental studies, with between 8 and 150 participants, examining the effects of valerian on sleep. These had been published

between 1982 and 2000 and, again, had inconsistent results.[11] Another recent systematic review found substantial heterogeneity in the estimated effect of valerian on sleep onset latency, with two studies favouring valerian and the third being well on the side of placebo.[8] The estimate for polysomnography (9.5 minutes, 95% CI -11.3 to 30.3) was different from the estimate for sleep diary (-16.0 minutes, 95% CI -29.5 to -2.5). The results for other outcomes in this review were not statistically significant, and there was also substantial heterogeneity in the estimated effect for sleep quality. The valerian studies included in this review did not show any significant difference in the number of adverse events reported for valerian compared with placebo, with an estimated risk difference of -0.06, in favour of valerian. Heterogeneity among studies was also substantial for this outcome. Eight randomised trials of valerian in adults published after 2000 are included in the Cochrane Central Register of Controlled Trials (2005, Issue 2) and Medline (PubMed searched 18 July 2005 using 'valerian' and publication type 'randomized controlled trial'). [13, 14, 15, 16, 17, 18, 19, 20] These studies provide further support for the safety of valerian, but the evidence of clinically important effects for people suffering from primary insomnia remains inconclusive, and the existence and size of any benefits remain uncertain. A more recently published trial conducted entirely using the Internet included 135 participants with both anxiety and insomnia in each group.[21] The valerian and placebo groups had similar improvements in sleep. Participants in both groups reported substantial improvements in all indices of sleep two weeks after starting either valerian or placebo. Adverse events in this study also occurred with similar frequency in the valerian and placebo groups.

In summary, many adults suffer from primary insomnia in Norway. There are a variety of remedies available, many of which are self-administered and can be used without the assistance of a health professional. The evidence of the effects of these remedies is, for the most part, inconclusive. We propose to rigorously evaluate the effects of a herbal over-the-counter drug with annual sales worth over 20 million crowns in Norway.[11] A control group will be given tablets identical to the valerian tablets, but which do not contain any valerian.

The study will be conducted as part of a collaboration between the Norwegian Knowledge Centre for the Health Services and the Norwegian Broadcasting Corporation (NRK) television program related to health issues, "Puls". The study will be used as an example of a clinical study in the explaining to the TV audience some of the principles related to clinical research.

2 OBJECTIVES

The primary objective of this trial is to evaluate whether valerian root (valerian) improves the self-assessed quality of sleep compared with placebo for people with primary insomnia.

The secondary objectives are to evaluate

- Latency to sleep onset
- Number of night awakenings
- Total sleep time
- Energy level in the following day

3 POPULATION

3.1 Inclusion criteria

1. Aged 18 –75 years, both inclusive
2. Insomnia lasting more than one month
3. PSQI score of > 5
4. Access to Internet and with own email address
5. At least 10 days of the sleep diary completed prior to randomisation

3.2 Exclusion criteria

1. Secondary insomnia
2. Use of hypnotics by prescription
3. Depression
4. Alcohol or drug abuse
5. Psychotherapy within the past six months
6. Sleep apnoea, periodic limb movements disorder or restless legs syndrome
7. Pregnant or lactating females or females of childbearing potential who do not use an approved method of contraception (oral contraceptives or IUD (Intrauterine device))
8. Shift workers
9. History of hypersensitivity to valerian or its constituents
10. Rating of 'usually' or 'always' to the following questions in the Global Sleep Assessment Questionnaire:
    During the past four weeks, how often:
    - Did you hold your breath, have breathing pauses, or stop breathing in your sleep?
    - Did you have restless or "crawling" feelings in your legs at night that went away if you moved your legs?
    - Did you have repeated rhythmic leg jerks or leg twitches during your sleep?
    - Did you have nightmares, or did you scream, walk, punch, or kick in your sleep?
    - Did any of the following disturb you in your sleep:
        - Pain?
        - Other physical symptoms?
        - Medications?
    - Did you snore loudly?
11. Current participation in another trial using an investigational compound

3.3 Recruitment and screening

Information about the study will be broadcasted during the Norwegian Broadcasting Corporation (NRK) television program, "Puls". Viewers interested in participation will be invited to visit the web pages of the study to enrol. If enough participants have not registered for the trial after two weeks, additional information will be broadcasted in another NRK "Puls" program to encourage more viewers to participate.

The initial screening of potential participants will be conducted online and will be automated, with options for potential participants to contact the study's Secretariat by email or telephone if they have questions for, or would like to discuss the study in more detail with, the investigators. Information about the study will be provided to all potential participants on the web pages. In order to participate, people interested will have to answer several questions to ensure that they have understood the study procedures and potential risks and benefits before they consent to participation.

If, after this, they agree to participate, they will verify their consent online.

We will monitor the Sleep Diary for serious side-effects and obtain information about potential serious adverse events if needed. The participants are informed that we are not providing health care, and they are informed that they need to contact their doctor if they get sick during the trial. A study physician is available on phone all through the trial, to answer questions and provide advice if needed.

3.4 Participant withdrawal

In accordance with the Declaration of Helsinki, the participants will be informed that they have the right to withdraw from the study at any time. However, unless safety issues occur, we plan to follow the participants for the entire duration of the study in order to analyse effectiveness and

safety variables also for those participants withdrawing from the study medication. The reason for any kind of withdrawal will be recorded.

There will be two main categories for withdrawals from the study:

Complete withdrawal (i.e. stopping investigational product and also continued effectiveness and safety evaluations)

Standard reasons for withdrawing from further participation in the study and from the follow-up visits may be:

- Participant's decision (withdrawal of consent to participate)
- Participant lost to follow-up

Withdrawals from investigational product (i.e. stopping investigational product, but continuing follow-up visits, including effectiveness and safety evaluations)

Standard reasons from withdrawing from taking further investigational product, but continuing follow-up visits and safety evaluations may be:

- Unacceptable adverse events
- Participant request
- Investigator's discretion
- Intercurrent illness

However, whenever a participant is withdrawn from the study, or for whatever reason will not complete the diary for the remaining period, a final study evaluation will be attempted for that participant (visit 4) - stating the reason(s) why the participant was withdrawn from the study. All documentation concerning the participant will be as complete as possible.

We will follow up withdrawals due to non-completion of the diary to obtain the reason for non-completion. The participant will be sent email reminders. Withdrawals due to intercurrent illnesses or adverse events will be fully documented in the case record form, with the addition of supplementary information if appropriate and available.

4 TREATMENT PROCEDURES

4.1 Assignment to treatment

There will be no physical visits at the investigator's office. However, as four of the study days will have additional questions or procedures as compared to the remaining "regular" days, these days are referred to as "visits".

Participants will at the inclusion visit (Visit 1), be assigned a study number, which will be the identification number for the participant throughout the study.

Participants who qualify for randomisation at Visit 2 will be allocated a randomisation number. These participants will then be assigned to one of two treatment groups according to a pre-determined randomisation scheme, using a computerised procedure. Randomisation numbers will be assigned sequentially according to a randomisation list. Numbers cannot be missed or substituted. Any errors concerning randomisation will be documented and explained by the investigator.

4.2 Assessments and procedures

4.2.1 Overview

Participants who fulfil all of the inclusion criteria and none of the exclusion criteria will be included in the study (Visit 1). As part of the registration of interest in the study, the participant must inform about their email address, mailing address and telephone number.

The participant will be contacted by the investigator by email and given a user name, which is the same as the user's e-mail address, and a password for the clinical trial web pages.

During the study the participant will be sent reminders by email for completion of the diary.

A structured sleep diary will be completed electronically every day during the whole study. At Visit 2 (Day 10) the selection criteria once more will be confirmed, and the participant's ability to complete the sleep diary will be assessed. If the participant still fulfils all selection criteria and has completed the sleep diary every day, he/she will be randomised to study treatment. Participants who at Day 14 still fulfil all selection criteria and have completed the sleep diary at least ten of the fourteen days will also be randomised to study treatment.

The study treatment will be shipped directly from a pharmacy to the participant, and the participant will start taking the treatment at Visit 3. The participant will confirm receipt of the treatment by entering the treatment number inscribed on the medicine box label into the Sleep Diary.

The treatment period will commence the first day that the participant enters the number of study medicine tablets that s/he took into the Sleep Diary and will last 14 days, at the end of which the participant will complete the Sleep Diary as usual plus three more questions. (Visit 4).

Participants will be asked to avoid using other medication for insomnia and to maintain their usual self-help strategies for their sleeping difficulties.

4.2.2 Concomitant chronic disease

The participant will be asked to state all concomitant chronic diseases. To ensure that none of the exclusion criteria are met, the participant will in addition be specifically asked whether any of the following diagnoses are present or have been in the past:

- Depression
- Sleep apnoea
- Periodic limb movements
- Restless legs syndrome
- Alcohol or drug abuse
- Insomnia secondary to other health problems, such as pain

4.2.3 Concomitant medication

See section 5.5, below.

4.2.4 Demographics

- Age
- Gender
- Education

4.2.5 PSQI

The Pittsburgh Sleep Quality Index (PSQI) will be used to screen potential participants for insomnia (Appendix 1).[22] The PSQI includes 19 self-reported items and uses an algorithm to derive a global score from components of sleep quality, sleep latency, sleep duration, habitual sleep efficiency, sleep disturbance, use of sleeping medication, and daytime functioning. Although there are several questions that request the evaluation of the person's roommate, these are not used in deriving the score. We will not include these questions. The PSQI has robust

psychometric characteristics related to general sleep quality and is widely applicable in adult populations. It has internal consistency and a reliability coefficient (Cronbach's alpha) of 0.83 for its seven components. Numerous studies using the PSQI have supported high validity and reliability. Scores range from 0 to 21. Higher scores indicate more severe sleep problems. A score of > 5 indicates a "poor" sleeper and is the minimum needed to be included in the trial.

The score is calculated as follows:

The following questions are to be answered:

During the past month,

1. When have you usually gone to bed? ___________
2. How long (in minutes) has it taken you to fall asleep each night? ___________
3. When have you usually gotten up in the morning? ___________
4. How many hours of actual sleep did you get that night? (This may be different than the number of hours you spend in bed) ___________

5. During the past month, how often have you had trouble sleeping because you…	Not during the past month (0)	Less than once a week (1)	Once or twice a week (2)	Three or more times a week (3)
a) Cannot get to sleep within 30 minutes				
b) Wake up in the middle of the night or early in the morning				
c) Have to get up to use the bathroom				
d) Cannot breathe comfortably				
e) Cough or snore loudly				
f) Feel too cold				
g) Feel too hot				
h) Have bad dreams				
i) Have pain				
j) Other reason(s), please describe, including how often you have had trouble sleeping because of this reason(s): ______________________________				
6. During the past month, how often have you taken medicine (prescribed or "over the counter") to help you sleep?				
7. During the past month, how often have you had trouble staying awake while driving, eating meals, or engaging in social activity?				
8. During the past month, how much of a problem has it been for you to keep up enthusiasm to get things done?				
	Very good (0)	Fairly good (1)	Fairly bad (2)	Very bad (3)
9. During the past month, how would you rate your sleep quality overall?				

Score calculation:

Component 1 #9 Score ______

Component 2	#2 Score (≤ 15 min (0), 16-30 min (1), 31-60 min (2), > 60 min (3)) + #5a Score (if sum is equal 0=0; 1-2=1; 3-4=2; 5-6=3)	______
Component 3	#4 Score (=>7(0), 6-<7(1), 5-<6 (2), <5 (3))	
Component 4	(total # of hours asleep) / (total # of hours in bed) x 100 > 85%=0; 75%-84%=1; 65%-74%=2; < 65%=3)	______
Component 5	# sum of scores 5b to 5j (0=0; 1-9=1; 10-18=2; 19-27=3)	______
Component 6	#6 Score	______
Component 7	#7 Score + #8 Score (0=0; 1-2=1; 3-4=2; 5-6=3)	______
Add the seven component scores together	Global PSQI Score	______

4.2.6 Sleep diary

Following the initial screening, potential participants will be asked to complete an electronic (web-based) sleep diary for four (two + two) weeks.

Five variables will be measured on a daily basis:

- Latency to sleep onset
- Number of night awakenings
- Total sleep time
- Quality of sleep
- Energy level the following day

These variables have been used and cited in the literature as being important measures of sleep.[12, 18, 23, 24, 25] The response options that are used are modified from those used by Coxeter and colleagues in the following ways: We have used seven categories for all five questions for consistency and to facilitate interpretation relative to what has been found to be a clinically important mean difference on 7-point scales.[26] We have added an additional category at the upper end of sleep latency and we have used half hour intervals for total sleep time under five hours to pick up what are likely to be meaningful differences for these two outcomes, based on clinical experience. Finally, a question on the level of perceived refreshment post-slumber is not included because feelings of grogginess are common after awakening and this question has not been found to be helpful in clinical practice.

In addition the diary will include questions about

- Adverse events
- Concomitant medication
- Number of tablets taken

An automated email message will remind participants who have not completed their sleep diary by 1 p.m. each day to do so.

4.2.7 Global assessment score

At Visit 4 each participant will be asked to indicate for each of the five listed variables and globally whether they perceived a change for the two weeks during which they took tablets compared to the two weeks before.

4.2.8 Adverse events

See section 6.3, below.

4.3 Scheduling of procedures

4.3.1 Visit 1 (Day 0; Screening)

- written informed consent
- control of inclusion and exclusion criteria
- concomitant medication
- PSQI

4.3.2 Visit 2 (Day 10; Randomisation)

- confirmation of inclusion and exclusion criteria
- assessment of sleep diary compliance
- randomisation
- shipment of study medication
- adverse events (daily registrations, sleep diary)
- concomitant medication (daily registrations, sleep diary)

4.3.3 Visit 3 (Day 15; Start of treatment)

- first day of study medication
- adverse events (daily registrations, sleep diary)
- concomitant medication (daily registrations, sleep diary)

4.3.4 Visit 4 (Day 28; End of treatment)

- global assessment score
- return of remaining tablets and/or study medication box
- adverse events (daily registrations, sleep diary)
- concomitant medication (daily registrations, sleep diary)

5 STUDY TREATMENT

5.1 Study treatment and comparator

The following treatment supplies will be used in the study:

Study treatment:
Coated Valerina Forte tablets (200 mg extract per tablet, 3 tablets corresponding to 3600 mg *Valeriana officinalis*).

Comparator:
Placebo: Coated tablets matching Valerina Forte in terms of appearance, smell and taste.

5.2 Supply, packaging, labelling, handling and storage

Participants who qualify for randomisation at Visit 2 will receive study medication for 2 weeks (3x20 tablets in blister package). To allow time for shipment, first treatment dose is not to be taken until Visit 3.

The study treatment will be labelled with information according to GMP annex 13 and local regulation.

The study treatment will be stored in a pharmacy at room temperature in a secure area protected from unintended use, and shipped to the participant upon confirmation from the investigator that the participant fulfils the randomisation criteria. The study treatment will be sent as a registered letter, requiring the participant to confirm receipt by signature. The participant must enter the randomisation code on the study treatment box to verify that s/he has received the correct package.

The pharmacy will maintain detailed drug dispensing lists.

5.3 Dosage and administration

The participants are to swallow 3 tablets every night.

The optimum dose of valerian is unknown.[12] The European Medicines Agency (EMEA) final proposal for a Core Data for *Valerianae radix* (http://www.emea.eu.int/pdfs/human/hmpc/001499en.pdf) recommends a single dose of 2 to 3 grams half an hour to one hour before bedtime with an earlier dose during the evening if necessary. Three tablets of Valerina Forte, a 200 mg extract (1:6) per tablet, correspond to 3600 mg *Valeriana officinalis*. This is slightly more than the dosage recommended by EMEA, but less than the maximum dose recommended by the manufacturer (4 tablets) and far below the maximum recommended daily dose of nine grams.

In previous randomised trials using repeated doses, the amount of extract taken per day has ranged from 450 to 1215 mg.[12] Single dose studies have used quantities ranging from 60 to 900 mg.[12] In two trials doses of 450 and 900 mg were compared with placebo. They each found both doses of valerian to be superior to placebo but not different to each other, suggesting that 450 mg may be a sufficient dose for an acute effect. The extraction ratio of these extracts was, however, not reported, so it is unclear how much *Valeriana officinalis* was administered, and it was found in N-of-1 trials that 450 mg (equivalent to 2 grams of dry root and rhizome) was not effective, perhaps due to that being a relatively small dose.[18] We are therefore testing a larger dose to ensure the best possibility of observing an effect, if valerian is effective, and because there seems to be no evidence of an increased risk of side effects with the higher dose. Up to now reports of adverse events in randomised trials of valerian have been scarce and those that have been reported have been mild and similar to those experienced with placebo.[8, 12, 18]

5.4 Duration of treatment

Participants will be treated for a maximum of two weeks.

5.5 Concomitant medication

Any medications (prescription only as well as OTC drugs) necessary for the participant's welfare that are not listed among the exclusion criteria (i.e. hypnotics), and which do not interfere with the effectiveness evaluations, are permitted given that they are carefully documented in the applicable diary page.

Investigational new drugs (i.e. drugs that are not marketed in the local market) will not be co-administered with the study medications during the entire period of the study.

5.6 Study medication accountability

The participants will each day record in their diaries the number of tablets taken. Upon completion of the study, the participants will be asked to return remaining tablets and the box to the pharmacy. 18 tablets should be remaining in case of 100 % compliance. A pre-paid, return-addressed envelope will be enclosed with the study medication.

Accountability forms will be completed by the pharmacy and compared with the information given by the participants in the diaries.

6 RESPONSE VARIABLES AND ENDPOINTS

6.1 Assessment of effectiveness

6.1.1 Primary effectiveness variable

- Proportion of participants in each group with an improvement in self-assessed quality of sleep of ≥ 0.5 units between the average score for the 2 weeks before and 2 weeks during treatment

6.1.2 Secondary effectiveness variables

- Proportion of participants in each group with an improvement of ≥ 0.5 units between the average score for the 2 weeks before and 2 weeks during treatment for each of the four variables:
  - sleep latency
  - number of awakenings
  - total sleep time
  - energy level during the day
- Mean changes in the five outcomes listed above.
- Global self-assessment

If a difference is seen between the treatment groups for the primary and secondary variables, the following explorative variables will be analysed.

- Proportion of participants in each group with any improvement in mean difference (i.e. all score changes > 0) between the average score for the 2 weeks before and 2 weeks during treatment for each of the five variables:
  - quality of sleep
  - sleep latency
  - number of awakenings
  - total sleep time
  - energy level during the day
- Difference in the profile of the five endpoints during the intervention period taking into account the baseline period, including a potential time effect.

6.2 Assessment of safety

Safety will be assessed by evaluation of registered adverse events, see section 6.3.

6.3 Adverse events and Serious Adverse Events

6.3.1 Definitions of Adverse Events

Seriousness

Adverse Events (AE): any undesirable experience occurring to a participant during a clinical study, whether or not considered related to the investigational study medication(s).
All Adverse Events must be recorded in the sleep diaries

Serious Adverse Events (SAE) are defined as: an adverse experience that is fatal, life-threatening, disabling or which results in-patient hospitalisation or prolongation of hospitalisation, a congenital anomaly or occurrence of malignancy.

Unexpected Adverse Event: an experience not previously reported in the product information sheet or similar documents.

Severity

Mild*:* the adverse event is transient and easily tolerated.
Moderate*:* the adverse event causes the patient discomfort and interrupts the patient's usual activities.
Severe*:* the adverse event causes considerable interference with the patient's usual activities, and may be incapacitating or life-threatening.

Relationship to study medication

Unrelated*:*
This category is applicable to those adverse events, which, after careful medical consideration at the time of evaluation, are judged to be clearly and incontrovertibly due to extraneous causes (disease, environment, etc.) and do not meet the criteria for study medication relationship listed under remote, possible or probable.

Unlikely*:*
In general, this category is applicable to those adverse events, which, after careful medical consideration at the time they are evaluated, are judged to be remotely related to the test study medication. An adverse event may be considered remote if, or when, for example: (i) it does not follow a reasonable temporal sequence from administration; (ii) it could readily have been produced by the patient's clinical state, environmental or toxic factors, or other modes of therapy administered to the patient; (iii) it does not follow a known response pattern to the suspected study medication; (iv) it does not reappear or worsen when the study medication is re-administered.

Possible (must have first two criteria):
This category is applicable to those adverse events, which, after careful medical consideration at the time they are evaluated, the connection with the test study medication administration appears unlikely but cannot be ruled out with certainty. An adverse event may be considered possible if, or when: (i) it follows a reasonable temporal sequence from administration; (ii) it could readily have been produced by the patient's clinical state, environmental or toxic factors, or other modes of therapy administered to the patient; (iii) it follows a known response pattern to the suspected study medication; (iv) it does not reappear or worsen when the study medication is re-administered.

Probable (must have first three criteria):
This category is applicable to those adverse events, which, after careful medical consideration at the time they are evaluated, the connection with the test study medication administration appears to, with a high degree of certainty, be related to the test study medication. An adverse event may be considered probable if: (i) it follows a reasonable temporal sequence from administration; (ii) it could not be reasonably explained by the known characteristics of the patient's clinical state, environmental or toxic factors, or other modes of therapy administered to the patient; (iii) it disappears or decreases on cessation or reduction in dose. There are important exceptions when an adverse event does not disappear upon discontinuation of the study medication, yet study medication-relatedness exists; e.g. bone marrow depression, fixed study medication eruptions, and tardive dyskinesias; (iv) it follows a known response pattern to the suspected study medication; (v) it reappears upon re-challenge.

Not assessable:
When it is not possible to assign the event to any of the criteria above categories.

6.3.2 Reporting of adverse events

As this is a clinical trial based on participant responses only, the adverse event reporting will be slightly different from other clinical trials.

The participant will report Adverse Events on a daily basis using the web-based sleep diaries. The participant will be specifically asked whether a physician is seen to evaluate the event. The primary physician may be contacted and asked to fill out a standard adverse event form The form must be faxed within 24 hours to the study investigators, who will evaluate whether the event should be reported to the Ethics Committee and the Regulatory Authorities within 7 or 15 days, as applicable. All Serious Adverse Events will be followed up until resolution (i.e. asymptomatic, stabilisation or death).

7 STATISTICAL METHODS AND DATA MANAGEMENT

7.1 Study design

Randomised, double-blind, parallel group, placebo controlled trial.

7.2 Estimation of sample size

Self-assessed quality of sleep has been selected as the main outcome for this trial. For each participant we will calculate a mean value for the quality of sleep for the two weeks prior to being randomized and a mean value for the two weeks after the medication period has started. The proportion of participants in each group with a change in mean score of 0.5 or more will be compared under the null hypothesis claiming no difference versus the alternative hypothesis claiming a difference:

$$H_0\text{: } p_{valerian} = p_{placebo} \quad \text{versus} \quad H_1\text{: } p_{valerian} \neq p_{placebo}$$

The table below shows the number of participants needed to reject the null hypothesis, depending on which proportion that can be anticipated for the two groups. Sample size calculations are based on Pearson Chi-square 2-sided test for two proportions, with a significance level of 0.05; power set to 80% (calculations performed with SAS, PROC POWER).

Proportion1	Proportion2	Actual Power	N Per Group
0.15	0.25	0.800	250
	0.26	0.801	211
	0.27	0.801	180
	0.30	0.802	121
	0.35	0.803	73
0.25	0.35	0.801	329
	0.40	0.800	152
	0.50	0.802	58
0.30	0.40	0.800	356
	0.50	0.800	93
	0.55	0.805	61
0.40	0.50	0.801	388

Proportion1	Proportion2	Actual Power	N Per Group
	0.53	0.800	230
	0.54	0.801	199
	0.55	0.800	173
	0.60	0.800	97

The table shows that the number to be included depends heavily on the estimates we use for the two proportions and which difference we consider important to reveal. By including about 250-275 participants in each group we would be able to reject the null hypothesis if the difference in proportions is about 10-15%.

7.3 Randomisation

The randomisation scheme (in a 1:1 ratio for active:placebo) will be generated using a computerised procedure. A complete randomisation list containing details of all participant numbers and study group will be stored as essential documentation in the Trial Master File (concealed until completion of analysis).

7.4 Blinding and Code Breaking Instructions

The participant card, which will be sent to participants along with the shipment of treatment, informing that the patient participates in a clinical trial, includes a 24-hour telephone number that may be contacted if the randomisation code must be broken due to a medical emergency. The phone number is also listed on the medication box. The code should be broken only if knowing the identity of the treatment allocation would influence the treatment of the patient.

The time, date and reason for breaking the code must be recorded in addition to the names of the physician requesting the unblinding and the person opening the code.

If a physician needs to break the code, the project management should, if possible, be contacted prior to breaking the code. In all cases, the project management must be notified within 24 hours after the code has been broken.

7.5 Statistical analysis plan

The randomisation code will be broken in two steps. The participants will be identified as belonging to group “a” or “b” without revealing which is the valerian and placebo group. The “a” and “b” code will not be broken until the primary and secondary statistical analyses have been completed and reviewed by the investigators.

All analyses will be done on an “intention-to-treat” basis; i.e. all participants who report receiving study drugs and taking them at least once will be included.

Descriptive statistics
Demographic data will be summarised by treatment group using means, minimums, medians, maximums and standard deviations for normally distributed variables and frequency counts and percents for categorical variables. This information will be used to describe the participants and compare the two groups with respect to these variables.

For each outcome (quality of sleep, latency to sleep onset, number of awakenings, total sleep time, energy level the following day) we will report the proportion of participants in each group with an improvement. In addition, the mean values will be calculated for each participant for both

the baseline period and the intervention period as well as the change in mean score per participant for each or these five outcomes. The changes will be summarized by means, minimums, medians, maximums, standard deviations and the associated 95% confidence intervals.

Safety analyses will include tabulation of type and frequency of all adverse events. Any serious adverse events will be reported with comprehensive narratives.

Primary analysis
The primary analysis will compare the proportion of participants reporting an improvement in mean score of 0.5 for the quality of sleep, by a chi-square test. We will report the difference in proportion and the associated 95% confidence interval.

Secondary analyses will comprise

- Comparison of the proportion of participants reporting an improvement in mean score of 0.5 for the other four outcomes (sleep latency, number of awakenings, total sleep time, and energy level during the day) by chi-square tests
- Comparison of the proportion of participants with any improvement for each of the five outcomes by chi-square tests
- Comparison of the proportion of participants reporting improvement on the global self-assessment using two cut off points (better or much better, and any improvement) by chi-square tests
- Comparison of the mean changes in the five outcomes by two-sample t-tests.
- Comparison of the proportion of participants in each group recording one or more adverse events during the intervention compared to the run-in period by MacNamars test and a chi-square test for the between group comparison
- An explorative analysis using repeated measures analysis of variance techniques in order to reveal any difference in the profile of the five endpoints during the intervention period taking into account the baseline period. This will also comprise a possibility to evaluate a time effect, e.g. to evaluate whether the effect is different after first day of treatment compared to a later day of treatment.

In addition, we will compare the relationship between a change of 0.5, which we have defined as the minimal clinically important difference, with self-assessed improvement by testing the association of a change of $\geq$ 0.5 on each of the 5 variables in the sleep diary with self-assessed improvement for all of the participants independent of which group they were in by chi-square tests.

The P values and confidence intervals will be reported without adjustments for multiple comparisons, and will be interpreted in the light of number of comparisons that are made.

7.6 Study populations

Intention-to-treat population
Participants will be included in the primary intention-to-treat population if they have entered the randomisation code of study treatment (available to them on study treatment packs only) and have documented that they have taken the study medicine at least once with a corresponding completion of the sleep diary.

Only the intention-to-treat population will be analysed in this study.

Data for all participants will be listed, and a list of withdrawn participants, with all reasons for withdrawal, will be given. This also applies to data for those participants who - after having consented to participate - underwent screening examinations required for inclusion into the study but who - because a criterion for exclusion was met or for other reasons - were not included in the study.

7.7 Data collection / case report forms

Data will be collected using an electronic, web-based sleep diary that participants will be asked to complete each day. The data entered into the diary will be automatically stored in a database. The participants email address and other identifying information will be kept in a separate database.

Participants will be asked to check that the information they enter is correct before saving it. The data will not be analysed until after the desired sample size has been reached and data has been collected for all of the included participants.

For serious adverse events, a person authorised by the investigators will enter information obtained from primary physicians into the database.

7.8 Data management

Automated validation checks will be set-up, and the participants will receive automated messages when entering inconsistent data or data that need follow-up or specific action.

Two databases will be set-up; one that includes only de-identified data of the participant (the participant is registered with participant number only) and one that can identify the participant (participant identification list) but that does not include sensitive data.

8 REGULATORY AND ADMINISTRATIVE PROCEDURES

8.1 Ethics committee

The study will be conducted in accordance with the Edinburgh, Scotland, (2000) amendment to the Declaration of Helsinki 1964.

The Protocol and Participant Information Leaflet and Informed Consent Form will be approved by the Ethics Committee before commencement. If a protocol amendment is necessary, this will be prepared with the agreement of the investigator, signed and submitted for ethical approval. Minor amendments that do not affect the safety or conduct of the study from the participant viewpoint, and which do not significantly reduce the scientific value of the protocol, and which do not require a significant change to be made to the consent form and/or the information sheet, will not be submitted for formal ethics review. These will be sent to the Ethics committee on an ‘information only’ basis.

The investigator is responsible for informing the ethics committees of any serious adverse events, as described in section 6.3.2.

8.2 Participant Information / Informed Consent

The investigator is responsible for giving the study participant full and adequate information about the nature, purpose, possible risk and benefit of the study. Study participants will also be notified that they are free to withdraw from the study at any time and that there is no risk associated with discontinuing to take the tablets. The participants can take as much time as they need to read and understand the information before signing (electronically).

Due to the web-based study design, only written information will be actively provided to the participant. Potential participants will be asked a series of questions to confirm that they

understand the information that is provided and will have the opportunity to contact the investigators by phone or email and ask questions before deciding whether they want to participate in the study.

The consent to study participation will be given electronically (online) at Visit 1. A second verification of interest in study participation will be obtained, as the medication will be sent as a registered letter, requiring the participant signature.

A copy of the participant information and of the Informed Consent Form in Norwegian, will be given to the participants both online and as paper-copy with the shipment of study medication, appendix 11.2.

As the study will be used for educational purposes for the TV-program audience with respect to clinical trial principles, journalists will follow some of the participants in the study. The participants will be asked upon inclusion whether they would be interested in being contacted by a journalist. It will be emphasized that this is not a prerequisite for participation and that only 4-6 participants will be contacted.

8.3 Participant confidentiality

The investigators must ensure that participant's confidentiality will be maintained. CRFs or other documents to be stored in the clinical trial database will only identify participants by their study number. The investigator will keep a separate log of participant codes and names. This database will also include the electronic confirmation of the participant's consent to study participation. The sleep diary will be in a secure zone. Each participant will be able to access her or his own sleep diary and no one else's. The investigators will only have access to anonymous data in tables. When serious adverse events are registered in the sleep diary, a message will automatically be sent to the investigators, who may then obtain additional information by contacting the participant and/or the primary physician, if needed.

Any patient medical records, received from the primary physicians in relation to adverse event reporting, will be reviewed by the study monitor and possibly by other sponsor personnel or regulatory authorities, to verify adequate source documentation, accuracy and completeness of Case Report Forms. The review will be conducted with strict adherence to professional standards of confidentiality (see section 8.8).

8.4 Participant treatment plan

After the individual completion of the study, the participant will be recommended to contact her primary physician for a thorough discussion about her insomnia and further treatment. Participants will be informed that, as soon as the study and analysis is completed, they will receive information by email on whether they have received placebo or valeriana.

8.5 GCP

The study will be managed and conducted according to the latest international (ICH) guidelines for Good Clinical Practice. The efforts of adapting the web-based study to comply with the guidelines are discussed throughout this protocol. As the participant will not meet health care professionals face-to-face as a consequence of study participation, the procedures for adverse event reporting and obtaining of informed consent are different from other clinical trials, at the same time striving to fulfil the requirements.

8.6 Essential documents

The ICH guidelines for GCP list a number of essential GCP documents required prior to, during and after the conduct of the study. A complete list of essential GCP documents can be found in the Investigator Site File.

8.7 Record retention

The CRFs and all medical records upon which the CRFs are based (source data) will be kept for at least 15 years after completion of the study. Image carriers or other data carriers may be used for this purpose.

8.8 Monitoring / Quality Control

The monitor will check study procedures, including safety assessments, study medication handling, data recording and source data verification (SDV) where applicable (the sleep diary will be the source of most data). The monitor will be allowed to review patient notes received from the primary physician in relation to adverse event reporting to confirm consistency between patient record and CRF data. Incorrect or missing entries into the CRFs will be queried and corrected immediately. Study monitoring will not jeopardise participant confidentiality.

8.9 Quality Assurance

During or after the study is completed, sponsor representatives or regulatory authorities may wish to carry out an audit. These representatives will have the same access to study data and participant source data as the monitor.

8.10 Insurance and Liability

Liability for study medication-induced injury will be according to Norwegian requirements.

8.11 End of Trial

The end of the trial is defined as the last visit of the last participant included in the trial. The competent authorities will be notified about the end of the trial.

8.12 Study report

A clinical study report will be prepared covering clinical and statistical aspects and summarising all findings of the clinical study.

8.13 Publication and Data Rights

The findings of the study will be published in a scientific journal.

The trial will be registered and assigned an ISRCTN prior to beginning recruitment. The results will be refereed by experts of insomnia and experts of the methods used in the trial before being published.

The published international guidelines for authorship (International Committee of Medical Journal Editors, 1997) will be adhered to; i.e. ‘All persons designated as authors should qualify for authorship. Each author should have participated sufficiently in the work to take public responsibility for the content.’

Authorship credit will therefore be based on substantial contributions to 1) conception and design, or analysis and interpretation of data; and to 2) drafting the article or revising it critically for important intellectual content; and on 3) final approval of the version to be published. Conditions 1), 2) and 3) must all be met. Participation solely in acquisition of funding or the collation of data does not justify authorship. General supervision of the research group is not sufficient for authorship. It is intended that information on what each author has contributed will be published.

After the results have been peer reviewed, the summarised results will be made available to participants in the trial and presented on the television program NRK “Puls”.

9 REFERENCES

1. Schenk CH, Mahowald MW, Sack RL. Assessment and management of insomnia. JAMA 2003; 289:2476-9.
2. Ohayon MM, Caulet M, Priest RG, Guilleminault C. DSM-IV and ICSD-90 insomnia symptoms and sleep dissatisfaction. Br J Psychiatry 1997; 171: 382–88.
3. Chevalier H, Los F, Boichut D, et al. Evaluation of severe insomnia in the general population: results of a European multinational survey. J Psychopharmacol 1999; 13 (4 Suppl 1): S21–24.
4. Sateia MJ, Nowell PD. Insomnia. Lancet 2004; 364:1959-73.
5. Hohagen F, Rink K, Käppler C, et al. Prevalence and treatment of insomnia in general practice: a longitudinal study. Eur Arch Psychiatry Clin Neurosci 1993; 242: 329–36.
6. Hatoum HT, Kania CM, Kong SX, Wong JM, Mendelson WB. Prevalence of insomnia: a survey of the enrollees at five managed care organizations. Am J Manag Care 1998; 4: 79–86.
7. Simon GE, VonKorff M. Prevalence, burden, and treatment of insomnia in primary care. Am J Psychiatry 1997; 154: 1417–23.
8. Buscemi N, Vandermeer B, Friesen C, Bialy L, Tubman M, Ospina M, Klassen TP, Witmans M. Manifestations and Management of Chronic Insomnia in Adults. Evidence Report/Technology Assessment No. 125. (Prepared by the University of Alberta Evidence-based Practice Center, under Contract No. C400000021.) AHRQ Publication No. 05-E021-2. Rockville, MD: Agency for Healthcare Research and Quality. June 2005.
9. Pallesen S, Nordhus IH, Nielsen GH, et al. Prevalence of insomnia in the adult Norwegian population. Sleep 2001; 24:772-9.
10. Holbrook AM, Crowther R, Lotter A, Cheng C, King D. Meta-analysis of benzodiazepine use in the treatment of insomnia. CMAJ 2000; 162:225-33.
11. Pallesen S, Bjorvatn B, Nordhus IH, Skjerve A. Valeriana as a sleeping aid: a literature review. Tidsskr Nor Lægeforen 2002; 122:2857-9.
12. Stevinson C, Ernst E. Valerian for insomnia: a systematic review of randomized clinical trials. Sleep Medicine 2000; 1:91-9.
13. Herrera-Arellano A, Luna-Villegas G, Cuevas-Uriostegui ML, et al. Polysomnographic evaluation of the hypnotic effect of Valeriana edulis standardized extract in patients suffering from insomnia. Planta Medica 2001; 67: 695-9.
14. Poyares DR, Guilleminault C, Ohayon NM, Tufik S. Can valerian improve the sleep of insomniacs after benzodiazepine withdrawal? Prog Neuropsychopharmacol Biol Psychiatry 2002; 26:539-45.
15. Glass JR, Sproule BA, Herrmann N, Streiner D, Busto UE. Acute pharmacological effects of temazepam, diphenhydramine, and valerian in healthy elderly subjects. J Clin Psychopharmacol 2003; 23:260-8.
16. Ziegler G, Ploch M, Miettinen-Baumann A, Collet W. Efficacy and tolerability of valerian extract LI 156 compared with oxazepam in the treatment of non-organic insomnia--a randomized, double-blind, comparative clinical study. Eur J Med Res 2002; 7:480-6.
17. Hallam KT, Olver JS, McGrath C, Norman TR. Comparative cognitive and psychomotor effects of single doses of Valeriana officianalis and triazolam in healthy volunteers. Human Psychopharmacology 2003; 18:619-25.
18. Coxeter PD, Schluter PJ, Eastwood HL, Nikles CJ, Glasziou PP. Valerian does not appear to reduce symptoms for patients with chronic insomnia in general practice using a series of randomised n-of-1 trials. Complement Ther Med 2003; 11:215-22.
19. Gutierrez S, Ang-Lee MK, Walker DJ, Zacny JP. Assessing subjective and psychomotor effects of the herbal medication valerian in healthy volunteers. Pharmacol Biochem Behav 2004; 78:57-64.

20. Diaper A, Hindmarch I. A double-blind, placebo-controlled investigation of the effects of two doses of a valerian preparation on the sleep, cognitive and psychomotor function of sleep-disturbed older adults. Phytother Res 2004; 18:831-6.

21. Jacobs BP, Bent S, Tice JA, Blackwell T, Cummings SR. An internet-based randomized, placebo-controlled trial of kava and valerian for anxiety and insomnia. Medicine 2005; 84:197-207.

22. Buysse D, Reynolds CF, Monk TH, Berman SR, Kupfer DJ. The Pittsburgh Sleep Quality Index: a new instrument for psychiatric practice and research. Psychiatry Research 1989; 28:193-213.

23. Plushner S. Valerian: Valeriana officinalis. Am J Health Syst Pharm 2000; 57:328–335.

24. Mills S,Bone K. The principles and practice of phytotherapy. Edinburgh: Churchill Livingstone,1999.

25. Leathwood PD, Chauffard F. Aqueous extract of valerian reduces latency to fall asleep in man. Planta Med 1985; April:144–8.

26. Guyatt G, Juniper E, Walter S, Griffith L, Goldstein R. Interpreting treatment effects in randomised trials. BMJ 1998; 316:690-3.

10 SIGNATURES

The protocol has been approved by:

Name and function	Signature	Date
Andrew D Oxman<br>Principal Investigator		
Signe Flottorp<br>Research Director		
Cheryl Carling<br>Researcher		
Atle Fretheim<br>Researcher		
Doris Tove Kristoffersen<br>Statistician / Researcher		
Torbjørn Fosen Wisløff<br>Statistician / Researcher		
Astrid Dahlgren<br>Research coordinator		
Ståle Pallesen<br>Associate professor		
Bjørn Bjorvatn<br>Professor		

11 APPENDICES

11.1 Study Flow-chart

**Phase**	**Observation period**		**Treatment period**	
**“Visit”**	**1**	**2**	**3**	**4**
**Day**	**0**	**10**	**15**	**28**
Selection criteria	X			
PSQI	X			
Informed consent	X			
Demographics	X			
Concomitant chronic diseases	X			
Concomitant medication	X	X	X	X
Confirmation of selection criteria		X		
Sleep diary compliance		X		
Randomisation / shipping of study treatment		X		
Confirmation of study treatment receipt/ first dose of treatment			X	
Global assessment score				X
Return study drugs				X
Adverse events	X	X	X	X
Sleep diary	<--->		<--->	

11.2 Patient Information/Informed Consent Form

Vær vennlig å svare på hvert av spørsmålene under *(det vil dukke opp en påminner på skjermen dersom du unnlater å svare på et spørsmål)*:

1. Jeg forstår at formålet med studien er å sammenlikne tabletter med urtemedisinen valeriana med tabletter som ikke inneholder valeriana, for å finne ut om tablettene med valeriana hjelper mot søvnproblemer.
   ☐ Ja ☐ Nei

2. Jeg forstår at informasjonen jeg gir, vil bli brukt til å finne ut om valeriana kan hjelpe folk som har søvnproblemer.
   ☐ Ja ☐ Nei

3. Jeg forstår at jeg kan stille spørsmål om forskningen før jeg bestemmer meg for å delta, og når som helst under studien, ved å kontakte sekretariatet for Søvnstudien.
   ☐ Ja ☐ Nei

4. Jeg forstår at jeg når som helst kan trekke meg fra studien, men at informasjonen jeg har gitt vil bli analysert uten at min identitet blir avslørt.
   ☐ Ja ☐ Nei

5. Jeg forstår at jeg vil bli spurt om jeg er villig til å la en journalist fra NRK Puls kontakte meg, men at jeg ikke behøver å svare ja til dette.
   ☐ Ja ☐ Nei

6. Jeg forstår at representanter for norske og utenlandske legemiddelmyndigheter (Statens legemiddelverk og lignende) kan få innsyn i relevante deler av min søvndagbok.
   ☐ Ja ☐ Nei

7. Jeg forstår at informasjonen jeg samler i min elektroniske søvndagbok vil bli brukt på en slik måte at jeg ikke kan bli identifisert, og at all informasjon behandles konfidensielt. Det vil si at ingen utenom prosjektgruppen og eventuelt representanter for legemiddelmyndighetene kan få vite at jeg har deltatt eller få kjennskap til noen annen personlig informasjon om meg uten min tillatelse.
   ☐ Ja ☐ Nei

8. Jeg forstår at jeg vil få automatiske påminnelser om å fylle ut søvndagboken til min e-postadresse.
   ☐ Ja ☐ Nei

9. Jeg forstår at hvis jeg fyller ut søvndagboken i 10 dager og er blant de 600 første som gjør dette, vil jeg motta tabletter i posten for at jeg skal bruke dem i to uker. Tablettene vil enten inneholde valeriana eller narremedisin (placebo). Jeg er kjent med at verken prosjektgruppen eller jeg vil vite hvilken av de to jeg har fått før etter at studien er avsluttet.
   ☐ Ja ☐ Nei

10. Jeg forstår at jeg skal fortsette å fylle ut søvndagboken mens jeg venter på tablettene og i to uker etter at jeg har mottatt tablettene.
    ☐ Ja ☐ Nei

Jeg har svart på alle spørsmålene, og jeg ønsker å starte en søvndagbok.
